# Action-Monitoring Dysfunction in Obstructive Sleep Apnea - A Pilot Study

**DOI:** 10.1371/journal.pone.0157575

**Published:** 2016-06-14

**Authors:** Ping-Song Chou, Chung-Yao Hsu, Meng-Ni Wu, Li-Min Liou, Shinag-Ru Lu, Ching-Kuan Liu, Chiou-Lian Lai

**Affiliations:** 1 Department of Neurology, Kaohsiung Medical University Hospital, Kaohsiung Medical University, Kaohsiung, Taiwan; 2 Department of Neurology, Kaohsiung Municipal Ta-Tung Hospital, Kaohsiung Medical University, Kaohsiung, Taiwan; 3 Department of Neurology, Faculty of Medicine, College of Medicine, Kaohsiung Medical University, Kaohsiung, Taiwan; 4 Department of Neurology, Kaohsiung Municipal Hsiao-Kang Hospital, Kaohsiung Medical University, Kaohsiung, Taiwan; University of Rome Tor Vergata, ITALY

## Abstract

Obstructive sleep apnea (OSA) is associated with a broad range of frontal lobe dysfunctions. However, no study has investigated action monitoring, a crucial domain of frontal cognitive functions, in patients with OSA. By using the modified Flanker task, we tested the hypothesis that patients with OSA have an impaired action monitoring function. We recruited 25 untreated patients with moderate–severe OSA and 12 control participants who were matched for age, sex, apolipoprotein E4, and education level. Every enrolled participant underwent a standard overnight laboratory-based polysomnography and completed a modified Flanker task. Compared with the controls, the patients with OSA presented a significantly lower correct response rate in all trials (78.9% vs 95.9%, *P* = .008), congruent trials (84.7% vs 98.3%, *P* = .016), and incongruent trials (77.4% vs 94.7%, *P* = .009). The post-error correction rate was significantly lower in the patients with OSA than in the controls (74.9% vs 93.8%, *P* = .005). Furthermore, strong significant correlations were observed between the arousal index and correct rate in all trials (*r* = −0.390, *P* < .05) and in the incongruent trials (*r* = −0.429, *P* < .01), as well as between the arousal index and rate of post-error correction (*r* = −0.435, *P* < .01). We concluded that the action monitoring function was impaired in the patients with OSA. Sleep fragmentation was a major determinant of impaired action monitoring in these patients.

## Introduction

Obstructive sleep apnea (OSA) is a common form of sleep-disordered breathing, and the prevalence of symptomatic OSA in middle-aged Asian men and women is 4.1%–7.5% and 2.1%–3.2%, respectively [1]. OSA, which is characterized by a repetitive narrowing or collapse of the pharyngeal airway during sleep, causes a cyclical breathing pattern with hypoxia and reoxygenation (intermittent hypoxia) and frequent brief awakenings from sleep (sleep fragmentation) [2]. Increasing evidence indicates that OSA is associated with hypertension [3], stroke [4], and cardiovascular mortality and morbidity [5]. Moreover, OSA has been associated with various neuropsychological problems such as depression [6] and neurocognitive impairment [7].

Based on frontal lobe pathology model of OSA, a metareview concluded that OSA affects frontal lobe function, particularly in the domains of attention/vigilance, verbal and visual delayed long-term memory, visuospatial/constructional ability, and executive function [7]. Beebe et al proposed that intermittent hypoxia and sleep fragmentation disrupt prefrontal cortical processes and cause deficits in frontal lobe function [8]. Intermittent hypoxia, which causes systemic inflammation and endothelial dysfunction, leads to changes in the microenvironment of neurons and affects synaptic plasticity [9, 10]. Sleep fragmentation, which disrupts normal sleep patterns, reduces the efficacy of restorative processes in the prefrontal cortex and leads to dysfunction of the neural networks in the frontal lobe [8].

Action monitoring, a crucial domain of frontal cognitive functions, plays a major role in adaptive behavior for detecting errors, preventing future errors, and hence optimizing individual performance. Humans tend to detect errors and then adjust their subsequent behavioral responses to avoid making more errors [11, 12]. Specific processes mediating a dedicated action monitoring system primarily center on the anterior cingulate cortex in the medial frontal lobe [13, 14].

Extended wakefulness and sleep deprivation can have deleterious effects on action monitoring [15, 16]. However, few studies have investigated the association between OSA and action monitoring. According to the frontal lobe pathology model of OSA, we hypothesize that patients with OSA have a higher tendency to make errors and a reduced ability to modify actions compared with people without OSA. Using the modified Flanker task, we investigated the action monitoring function of patients with OSA and whether intermittent hypoxia or sleep fragmentation is the major determinant of this function.

## Materials and Methods

### Participants

This study adopted a cross-sectional case–control study design. Patients with newly diagnosed and untreated moderate–severe OSA (apnea–hypopnea index, [AHI]>15) were recruited from Kaohsiung Medical University Hospital in Southern Taiwan. Control participants were recruited from the general population. OSA was diagnosed through complete overnight polysomnography (PSG). To exclude participants with possible OSA from the control group, all controls underwent an overnight PSG to ensure that their AHI was <5. All participants underwent a comprehensive medical evaluation including a review of their medical history, a physical examination, tests for blood chemistry, apolipoprotein E (APOE) polymorphism [17], metabolic syndrome [18, 19], and a cognitive assessment. We excluded participants with a history or clinical evidence of neurological or psychiatric disease, those receiving medical therapy that might affect their cognitive function (eg, a prescription of antipsychotics or sedatives), and those with scores below the cutoff value (adjusted by age and educational level) of the Cognitive Abilities Screening Instrument [20, 21].

This study was conducted in accordance with the Helsinki Declaration and was approved by the Institutional Review Board of Kaohsiung Medical University Hospital. Written informed consent was obtained from all the participants.

### Polysomnography

All participants underwent standard overnight laboratory-based PSG by using two validated machines (Nicolet Ultrasom and Respironic Alice 5). Physiological parameters were measured and recorded, including pulse oximetry, an electroencephalogram, an electrooculogram, nasal and oral air flow measurements, chest wall movements, an electromyogram, and an electrocardiogram. Sleep-related respiratory events were scored according to the American Academy of Sleep Medicine criteria [22]. The definition of hypopnea is that the peak signal excursions drop by ≥30% of the pre-event baseline when nasal pressure is applied for ≥10 seconds with ≥4% oxygen desaturation or the peak signal excursions drop by ≥50% for ≥10 seconds with ≥3% oxygen desaturation or associated arousal. Oxygen desaturation index (ODI) is defined as the number of ≥3% arterial oxygen desaturation events per hour during sleep. We recorded respiratory parameters including the AHI, ODI, arousal index, lowest blood pulse oxyhemoglobin saturation (SpO_2_), and percentage of time of SpO_2_ below 90%.

### Sleep questionnaire

All participants completed a self-report sleep questionnaire. A validated Chinese version of the Epworth Sleepiness Scale (ESS) was employed to evaluatie day-time sleepiness [23].

### Modified Flanker task

**Fig 1:**
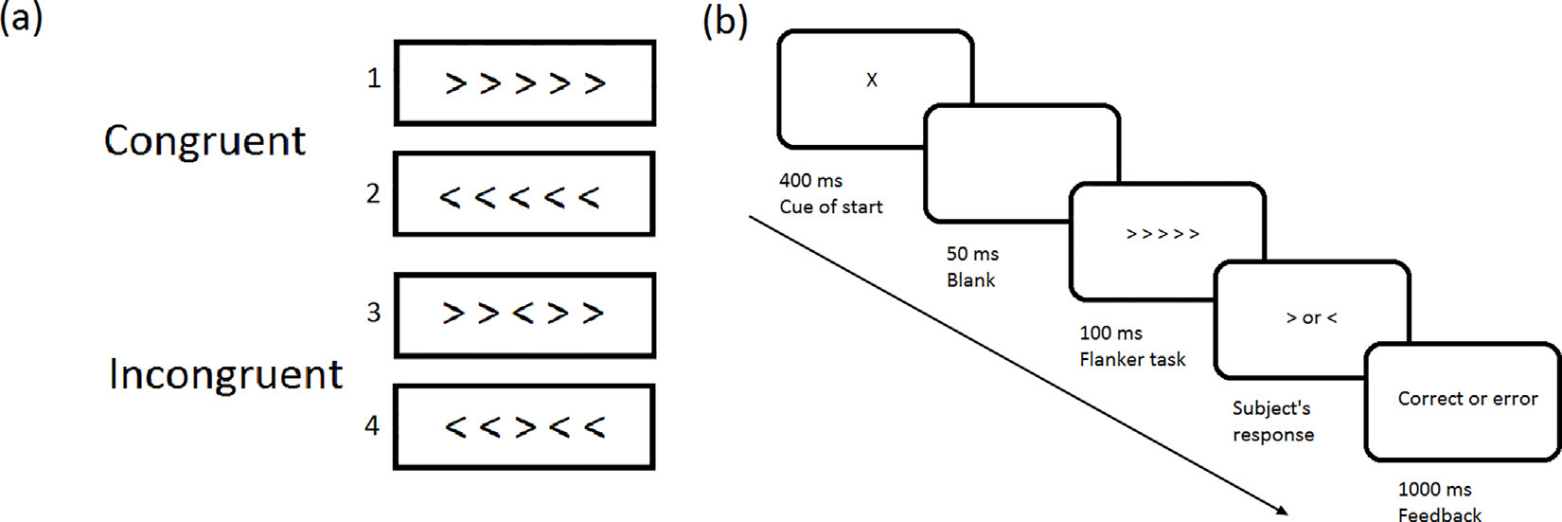
(a) Congruent (1,2) and incongruent (3,4) modified Flanker tasks and (b) modified Flanker task procedure.

E-Prime software 1.1 (Psychology Software Tools Inc., Sharpsburg, NC, USA) was used for the experimental procedure. The modified Flanker task [24] was applied to all participants in the morning after they completed the overnight PSG. Five visual stimuli were presented in the middle of a computer monitor. The participants were instructed to indicate the direction of a central target stimulus flanked by nontarget stimuli. In congruent trials, the nontarget stimuli corresponded to the same direction as the target (<<<<< or >>>>>), whereas in incongruent trials, the nontarget stimuli corresponded to the direction opposite that of the target (<<><< or >><>>) (Fig 1A). An accurate response was counted when the participants pressed a button on the keyboard corresponding to the direction of the center target. Participants were seated approximately 70 cm from the computer monitor and were instructed to use only their index fingers to respond. Finally, visual feedback was provided to all the participants according to the accuracy of their responses. No participant consumed alcohol, caffeine, nicotine, or any cognitive-enhancing medication at the time of testing.

In each trial, an X cue was presented for 400 ms, with the stimulus shown after another 50 ms the blank screen. The presentation of the stimulus was 100 ms and visual feedback was presented for 1000 ms after the stimulus. Both speed and accuracy of the responses are encouraged by a feedback (Fig 1B). An initial practice session involving 25 trials was conducted to ensure that all the participants understood the instructions. The formal task involved 5 sessions, each comprising 100 trials. The formal single session lasted for 3–4 minutes depending on how quickly the participants responded to the stimuli. A 30-second break was allowed between sessions. The stimuli in each formal section were presented in a random order with a 20% and 80% probability for congruent and incongruent trials, respectively. The order of stimuli differed in each session.

### Statistical analysis

Statistical analysis was performed using SPSS. All statistical tests were 2-tailed, and an alpha value of 0.05 was considered to indicate statistical significance. The Student *t* test was conducted for continuous variables, and the chi-squared test was used for categorical variables for determining the variation in demographic data, APOE4 prevalence, and PSG parameters between the patients with OSA and the controls.

The Student *t* test was conducted to evaluate the differences in the performance of the modified Flanker task between the patients with OSA and the controls. To study the association between the measures of the modified Flanker task and PSG parameters, Pearson correlation coefficients were used, and the level of significance was defined as *P* < .05.

## Results

### Demographic characteristics and PSG parameters

Twelve healthy adults and 25 patients with OSA participated in the study. Among the patients with OSA, 19 (76%) were men with a mean age of 49.7 ± 8.6 years (30–62 y). Among the controls, 6 participants (50%) were men with a mean age of 43.9 ± 8.7 years (32–62 y). Participant characteristics, including demographic data, the prevalence of metabolic syndrome, and APOE4 are presented in Table 1. Demographic data, namely age, sex, education level, the prevalence of metabolic syndrome, and APOE4, did not differ significantly between the 2 groups (Table 1).

**Table 1:** Demographic characteristics, parameters of polysomnography, and ESS of the OSA patients and controls.

	Patients with OSA (n = 25)	Controls (n = 12)	*p* value
Gender, male, n (%)^1^	19 (76.0)	6 (50.0)	0.146
Age, year, mean (±SD)^2^	49.7 (±8.6)	43.9 (±8.7)	0.065
Education, year, mean (±SD)^2^	13.4 (±2.3)	14.3 (±2.6)	0.349
Metabolic syndrome, n (%)^1^	12 (48.0)	2 (16.7)	0.084
APOE4, n (%)^1^	6 (27.3)	7 (58.3)	0.139
Apnea-Hypopnea index, /hour, mean (±SD)^2^	29.3 (±15.3)	1.2 (±1.6)	0.000
Oxygen desaturation index, /hour, mean (±SD)^2^	25.6 (±16.3)	0.8 (1.1)	0.000
Arousal index, /hour, mean (±SD)^2^	23.6 (±14.6)	8.6 (±5.6)	0.000
Lowest sleep SpO2, %, mean (±SD)^2^	76.2 (±8.2)	84.0 (±11.2)	0.021
SpO2 < 90%, %, mean (±SD)^2^	10.5 (±14.7)	2.5 (±4.0)	0.075
ESS, mean (±SD)^2^	10 (3.8)	7 (2.5)	0.020

OSA: obstructive sleep apnea; SD: standard deviation; APOE4: apolipoprotein E4; SpO2: blood oxygen saturation; ESS: Epworth Sleepiness Scale

^1^: Chi-square test, versus controls

^2^: Student *t* test, versus controls

Among the patients with OSA, the mean AHI, ODI, and arousal index were 29.3 ± 15.3, 25.6 ± 16.3, and 23.6 ± 14.6, respectively. The mean ESS was 10.0 ± 3.8. Patients with OSA had significantly higher AHI, ODI, arousal index, and ESS, and lower lowest blood SpO_2_ compared with the controls (Table 1).

### Action monitoring: Performance on the modified Flanker task

**Fig 2:**
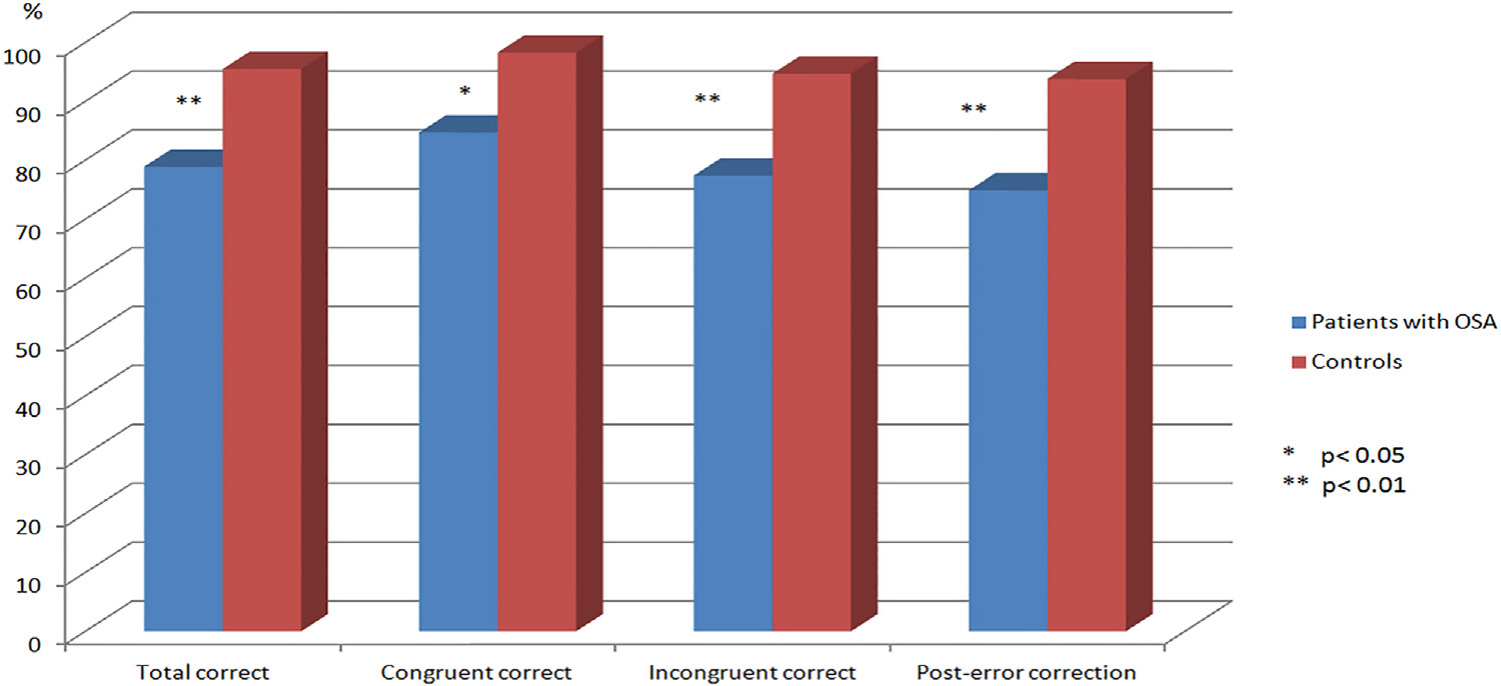
The differences of performance on the modified Flanker task between patients with OSA and controls.

Results and statistics of the modified Flanker task completed by all the participants are summarized in Table 2. Patients with OSA had a significantly lower correct response rate in all the trials (78.9% vs 95.9%, *P* = .008), congruent trials (84.7% vs 98.3%, *P* = .016), and incongruent trials (77.4% vs 94.7%, *P* = .009) compared with the controls. To assess the post-error behavioral adjustments, post-error correction was defined as a correct response following an erroneous response. The post-error correction rate in the patients with OSA was significantly lower than that in the controls (74.9% vs 93.8%, *P* = .005) (Fig 2).

**Table 2:** Flanker task performance in OSA patients and controls.

	Patients with OSA (n = 25)	Controls (n = 12)	*p* value
Correct, %, mean (±SD)	78.9 (±28.3)	95.5 (±3.8)	0.008
Congruent	84.7 (±25.9)	98.3 (±3.6)	0.016
Incongruent	77.4 (±30.2)	94.7 (±4.1)	0.009
Post-error correction, %, mean (±SD)	74.9 (±28.7)	93.8 (±8.3)	0.005
Reaction time, ms, mean (±SD)			
Correct	386.3 (±84.8)	392.1 (±51.7)	0.827
Congruent	349.0 (±76.5)	341.2 (±45.7)	0.748
Incongruent	394.0 (±91.1)	405.2 (±54.8)	0.698
Post-error correction	405.5 (±99.2)	427.3 (±66.7)	0.496

OSA: obstructive sleep apnea; SD: standard deviation. P value: Student *t* test, versus controls

During the modified Flanker task, no significant difference was observed between the patients with OSA and the controls regarding the reaction time in all the correct trials, correct congruent trials, correct incongruent trials, and post-error corrections.

### Correlations of action monitoring and PSG parameters/ESS

The performance on the modified Flanker task was analyzed for correlations with the PSG parameters and ESS. Pearson *r* coefficients between the performance on the modified Flanker task and PSG parameters/ESS are listed in Table 3.

**Table 3:** Correlation between Flanker task and PSG parameters.

	Arousal index (/hr)	AHI (/hr)	ODI (/hr)	SpO2<90% (%)	Lowest SpO2 (%)	ESS
Correct, %	-0.390*	-0.251	-0.231	-0.104	0.008	0.022
Congruent	-0.134	-0.257	-0.231	-0.127	0.028	0.088
Incongruent	-0.429**	-0.240	-0.222	-0.095	0.003	0.007
Post-error correction, %	-0.435**	-0.327*	-0.297	-0.189	0.064	0.001
Reaction time (ms)						
Correct	-0.072	0.057	0.133	0.234	-0.289	-0.202
Congruent	-0.012	0.101	0.164	0.240	-0.253	-0.153
Incongruent	-0.147	0.050	0.123	0.234	-0.296	-0.207
Post-error correction	-0.184	-0.030	0.028	0.201	-0.302	-0.168

PSG: polysomnography; OSA: obstructive sleep apnea; REM: rapid eye movement; AHI: apnea-hypopnea index; ODI: oxygen desaturation index; hr: hour; SpO2: blood oxygen saturation; ESS: Epworth Sleepiness Scale

Pearson *r*

* *P* < .05

** *P* < .01, by Pearson correlation

We considered the AHI, arousal index, and ODI as indicators of OSA severity, sleep fragmentation, and intermittent hypoxia, respectively. Significant correlations were observed between the arousal index and correct rate of total trials (*r* = −0.390, *P* < .05) and incongruent trials (*r* = −0.429, *P* < .01) of the modified Flanker task. Moreover, the post-error correction rate correlated significantly with the arousal index (*r* = −0.435, *P* < .01) and AHI (*r* = −0.327, *P* < .05). No significant correlations were observed between the performance in the modified Flanker task and the ODI, lowest blood SpO_2_, percentage of time of SpO_2_ below 90%, and ESS.

## Discussion

According to our review of relevant literature, this study is the first to examine the action monitoring function of patients with OSA. We employed the modified Flanker task to investigate the action monitoring function of patients with OSA. Our results reveal that these patients typically made more errors in the trials and were less accurate following an incorrect response, suggesting impairment in action monitoring function. The patients with OSA exhibited a deficiency in corrective behavior. The correlation coefficients suggest that the impairment of action monitoring function is mainly associated with sleep fragmentation.

Action monitoring plays a major role in adaptive behavior for detecting errors, preventing future errors, and hence optimizing individual performance. Tulek et al used the Flanker task to disclose partially impaired attentional control in patients with OSA; however, their post-error analysis yielded nonsignificant results, and corrective behavior was not assessed [25]. Using the Flanker task, an experimental study on sleep deprivation revealed that post-error adjustments were maintained after one night of complete sleep deprivation [26]. In another similar study conducted by Renn et al, Flanker task results revealed mild impairment in error monitoring behavior [27]. However, experimental situations of sleep deprivation cannot completely reflect the precise pathophysiology of OSA. Our study results suggest that patients with OSA have impaired action monitoring function and could not adjust their behaviors when they made errors during the modified Flanker task.

Error-associated behavioral modifications are mediated by the frontal lobe [28], and frontal lobe dysfunction has been associated with OSA. Based on cerebral structure magnetic resonance imaging (MRI), significant reductions in gray matter in the anterior cingulate, hippocampus, frontal, parietal, and temporal lobes were found to correlate with OSA severity [29]. Furthermore, diffusion tensor imaging revealed impaired white matter integrity in multiple brain areas, namely the deep frontal, medial, prefrontal, and parietal lobes of patients with OSA [30]. Using functional MRI, Thomas et al reported that patients with OSA exhibited reduced activation in the anterior cingulate, dorsolateral prefrontal, and posterior parietal cortices [31]. Thus, structural change and functional disturbance in the frontal–cortical networks in patients with OSA appear to be associated with multiple cognitive dysfunctions [30, 32, 33] and may also contribute to action monitoring impairment, which centers on the anterior cingulate cortex.

Our second main finding is that sleep fragmentation is a major determinant of action monitoring impairment. In our study, the performance on the modified Flanker task correlated significantly with the arousal index but not with the ODI or nocturnal hypoxia. The possible mechanism is that sleep fragmentation, which disrupts normal sleep patterns, reduces the efficacy of restorative processes and leads to neural network dysfunction in the frontal lobe [8].

Several studies involving the use of functional imaging and experimental sleep fragmentation support this finding. Ko et al reported a trend of reduced post-error accuracy in the Flanker task in high-level experimental sleep fragmentation compared with low-level experimental sleep fragmentation [34]. Two studies using functional imaging have concluded that sleep fragmentation possibly contributed more to decreased brain activation in the cingulate, frontal, and parietal regions than did nocturnal hypoxia in patients with OSA [31, 35]. Together with our findings, decreased activity in the frontal lobe can contribute to action monitoring impairment in patients with OSA, and sleep fragmentation plays a major role in this pathophysiology.

To the best of our knowledge, our study is the first to investigate action monitoring function and reveal impaired corrective behavior in patients with OSA. However, our study has some limitations. First, because of the cross-sectional study design, our results are preliminary. Future studies with a long-term follow-up after continuous positive airway pressure treatment should be conducted. Second, considering higher ESS scores, the impaired action monitoring could be attributed to sleepiness in OSA patients. Although the performance of the Flanker task was poor in the OSA patients, the reaction time in the Flanker task did not differ significantly between the OSA patients and controls. In addition, the correlation between ESS and the results of the Flanker task was low. Therefore, sleepiness might not play a crucial role in action monitoring in OSA. Third, our study reveals that the arousal index, which is commonly used as an indicator of sleep fragmentation, was correlated significantly with action monitoring impairment in patients with OSA. An elevated arousal index might be due to respiratory events including apnea, hypopnea, and respiratory effort-related arousals, periodic limb movement in sleep, or the involvement of spontaneous arousal desaturation. Thus we cannot exclude that action monitoring is impaired due to sleep fragmentation and OSA is a major cause of sleep fragmentation. Future studies investigating other sleep disorders with sleep fragmentation but without hypoxia, such as insomnia or periodic limb movement disorder, may assist in clarifying this issue.

## Conclusions

In conclusion, action monitoring function, particularly in corrective behavior, appears to be impaired in patients with OSA. Sleep fragmentation is a major determinant of action monitoring impairment in such patients. Our findings reveal that the modified Flanker task is an easy and sensitive procedure for evaluating action monitoring function in patients with OSA.
